# Asymptotics of the meta-atom: plane wave scattering by a single Helmholtz resonator

**DOI:** 10.1098/rsta.2021.0383

**Published:** 2022-11-28

**Authors:** M. J. A. Smith, P. A. Cotterill, D. Nigro, W. J. Parnell, I. D. Abrahams

**Affiliations:** ^1^ Department of Applied Mathematics and Theoretical Physics, University of Cambridge, Wilberforce Road, Cambridge CB3 0WA, UK; ^2^ Department of Mathematics, The University of Manchester, Oxford Road, Manchester M13 9PL, UK; ^3^ Thales United Kingdom, 350 Longwater Avenue Green Park, Reading RG2 6GF, UK

**Keywords:** acoustics, Helmholtz resonator, scattering, matched asymptotic expansions, multipole methods

## Abstract

Using a combination of multipole methods and the method of matched asymptotic expansions, we present a solution procedure for acoustic plane wave scattering by a single Helmholtz resonator in two dimensions. Closed-form representations for the multipole scattering coefficients of the resonator are derived, valid at low frequencies, with three fundamental configurations examined in detail: the thin-walled, moderately thick-walled and extremely thick-walled limits. Additionally, we examine the impact of dissipation for extremely thick-walled resonators, and also numerically evaluate the scattering, absorption and extinction cross-sections (efficiencies) for representative resonators in all three wall thickness regimes. In general, we observe strong enhancement in both the scattered fields and cross-sections at the Helmholtz resonance frequencies. As expected, dissipation is shown to shift the resonance frequency, reduce the amplitude of the field, and reduce the extinction efficiency at the fundamental Helmholtz resonance. Finally, we confirm results in the literature on Willis-like coupling effects for this resonator design, and connect these findings to earlier works by several of the authors on two-dimensional arrays of resonators, deducing that depolarizability effects (off-diagonal terms) for a single resonator do not ensure the existence of Willis coupling effects (bianisotropy) in bulk.

This article is part of the theme issue ‘Wave generation and transmission in multi-scale complex media and structured metamaterials (part 2)’.

## 1. Introduction

*Wave scattering* is a process describing how an incident wave is modified by the presence of an object, or ensemble of objects, in its path [1,2]. Factors such as the geometry of the scatterer or incident angle of the wave can induce major changes in the observed response, and using rigorous mathematical analysis, it is possible to provide insight on a diversity of observable effects and real-world phenomena. For example, wave scattering can be used to identify delaminations in composite media with ultrasound [3], and explains how water droplets in the air can give rise to vibrant rainbows [1]. This broad definition of scattering applies to waves of all types, from surface waves on deep water through to electromagnetic, elastic and sound waves. In general, there are two canonical geometries whose scattering properties are now generally well-understood: spheres and idealized (infinitely long) circular cylinders.

There has been extensive research attention on wave scattering by spheres since at least the works of Mie [4], whose thorough outline of the separation of variables solution for the vector Helmholtz equation continues to influence scientific research well into the present day [1,3]. This is due to the fact that suspensions of spherical, or approximately spherical, objects arise in an extensive range of applications, from the homogenization of milk to air pollution modelling. Wave scattering by infinitely long circular cylinders has likewise received considerable attention, due to its extensive real-world applications, from the design of fibrous materials such as kevlar to aerofoil design for aircraft [3]. It would appear that the first comprehensive multipole solution for scattering by circular cylinders, in the setting of Maxwell’s equations, was conducted by von Ignatowsky [5], although the first scattering solution was outlined some decades earlier by Lord Rayleigh [6]. An exhaustive early history of the scattering literature for spheres and cylinders may be found in Kerker [2]. Interest in multipole solutions for cylinders has grown significantly in recent decades with the advent of two-dimensional photonic, phononic and other crystals as well as with the advent of metamaterials (discussed below) [7,8]; however, results have been primarily obtained using numerical methods, with few analytical treatments available for non-circular cylindrical configurations.

**Figure 1.**
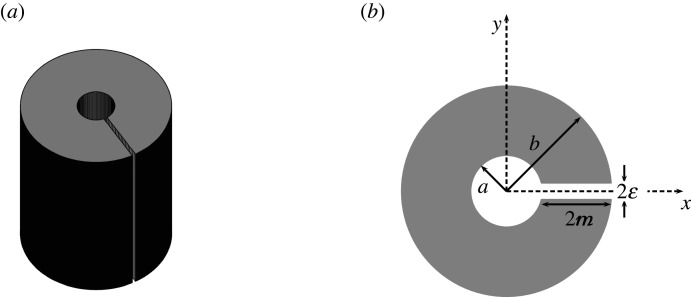
(*a*) Three-dimensional segment and (*b*) two-dimensional cross-section of our infinitely long Helmholtz resonator in dimensionless coordinates, where we define the outer radius b, the total aperture width 2ε, the total aperture length 2m and the inner radius a=b−2m.

In this work, we examine an important extension to scattering by an infinitely long cylinder: we consider two-dimensional acoustic plane-wave scattering by a circular cylinder into which we have introduced a hollow circular cavity and neck to form a Helmholtz resonator, where Neumann boundary conditions are imposed on all walls. A representative resonator is presented in figure 1 for guidance. Using multipole methods and the method of matched asymptotic expansions [9,10], we construct a low-frequency, closed-form solution to describe the scattering coefficients for the resonator and the resulting potential field in the exterior domain. We are unaware of any other works in the literature which present low-frequency analytical expressions for the scattering coefficients of Helmholtz resonators in this manner. That said, scattering by a single thin-walled elastic Helmholtz resonator shell in a fluid has been considered previously [11] and their resonance condition (eqn. (C27)) in the rigid limit is equivalent to ours in the thin-walled setting. Also, matched asymptotic expansions have previously been used to characterize plane wave scattering by a semi-cylindrical resonator embedded in the boundary of a half-space [12]. Here, we consider three resonator geometries explicitly: the thin-walled, moderately thick-walled and extremely thick-walled cases, providing closed-form expressions and compact asymptotic forms in all instances.

A key objective of the present work is to obtain insight on wave propagation through more complex arrangements of resonators, for example, when they are tiled periodically to form finite clusters, gratings, or lattices [13,14]. The scatterer considered here is a canonical geometry for two-dimensional metamaterials, and following an established nomenclature in the literature (i.e. Melnikov *et al.* [15]) our resonator is considered to be a ‘meta-atom’. This terminology arises from the fact that a two-dimensional periodic lattice of such resonators can give rise to a *metamaterial*, a type of composite material that exhibits unusual and unexpected properties that are not readily observed in conventional media [7,8].

In addition to our multipole-matched asymptotic solution, we also compute *cross-sections* for a single cylindrical Helmholtz resonator. These are measures of strength for different wave processes, for example, describing how much incident power is scattered or absorbed by the resonator. We also evaluate extinction cross-sections, which refer to the power loss in the downstream direction to the incident field (where a detector or observer would be located), due to the presence of the object [1]. Formally, extinction is defined as the sum of both absorption and scattering processes, and in particular frequency regimes, or for certain geometries, either scattering or absorption may dominate [1, ch. 11]. In this work, we consider cross-sections that are non-dimensionalized by the diameter of a closed cylinder and we term the resulting quantities *efficiency coefficients*. Note that the extinction cross-section may be used to determine an estimate for the absorbance (attenuance) of a large random ensemble of resonators; this is obtained by multiplying the extinction cross-section by the filling fraction and the mean path length through the cluster (i.e. following the Beer–Lambert Law [16]). A substantial body of the literature has been dedicated towards cross-sections, particularly for spherical and cylindrical geometries, giving rise to significant variations in definitions [1,17–21]. In order to avoid any possible confusion, and to facilitate comparison with the literature, we present results for closed (ideal) Neumann cylinders where possible.

To further complement the above, we briefly examine the impact of dissipation in our acoustic resonator. As discussed in the literature [22,23], there are a range of diffusive and dissipative mechanisms present in all acoustic systems, such as bulk fluid thermal/viscous effects, radiation damping losses, boundary layer effects, vortex shedding and flow separation. In this work, we consider a fully developed boundary layer in the neck of extremely thick-walled resonators only, as we consider this to be the dominant internal loss mechanism [24]. A boundary layer refers to the region close to a surface where thermal or viscous effects within the fluid become strong, ultimately dissipating wave energy. There are a variety of ways of taking into account such losses in a duct, e.g. the full thermo-visco-acoustic equation [24] or via a lossless wave equation for the pressure field with complex admittance boundary conditions on the two walls [25,26]. For the lowest-order propagating mode, this typically leads to a frequency-dependent absorption coefficient that scales with the square root of the frequency; however, it is assumed that this dependency will not affect the results significantly here (over the frequency range of interest) and thus for simplicity, we will take the absorption coefficient to be constant. We are able to show numerically that such dissipation generally reduces the resonant enhancement in the scattering and extinction cross-sections (efficiencies) to recover results for a closed Neumann cylinder, with the absorption efficiency exhibiting a clear peak as the attenuation coefficient $\bar{\alpha }$ increases. This absorption peak implicitly defines the operating range of our model, and we do not advise considering $\bar{\alpha }$ values beyond this range, although in the limit $\bar{\alpha }\to \infty$ results must return to those for a closed Neumann cylinder, as the ever-increasing resistance in the neck region inhibits energy flow to the interior of the resonator.

We remark that our closed form (multipole) solution representation should prove useful to those interested in the scattering behaviour of resonators, as our expressions are rapidly evaluable with little computational overhead. Our methodology avoids the need to use intensive numerical methods (where meshing in the narrow neck region can become prohibitive), and permits the rapid exploration of parameter spaces for optimization. To this end, we present closed-form representations for the dispersion equation corresponding to the first Helmholtz resonance, in all three wall thickness regimes. In the extremely thick-walled regime, the fundamental Helmholtz resonance can be pushed to very low frequencies by tuning the outer radius, neck width and neck length carefully (see Smith & Abrahams [14]).

The outline of this paper is as follows. In §2, we present a brief solution outline for plane wave scattering by a closed (ideal) Neumann cylinder. Next, we pose an ansatz for the total potential of a single resonator satisfying Neumann conditions on the scatterer’s walls (which is known except for an undetermined coefficient) in §3. In §3b, we use matched asymptotic expansion methods to determine this amplitude for resonators in the thin-walled, moderately thick-walled and extremely thick-walled regimes. Closed-form expressions for determining the Helmholtz resonance frequency are then derived and presented for these three settings in §3c. In §4, asymptotic representations for the scattering coefficients and definitions for the cross-sections are given, with dissipation in extremely thick-walled cylinders considered in §5. Numerical results are presented in §6 and are followed by concluding remarks in §7. Finally, in appendix A, we discuss and clarify Willis-like coupling effects that have been reported in the literature for plane-wave scattering by resonators of this or similar design.

## 2. Scattering by a single cylinder

As a means of reference, we begin by briefly outlining the multipole solution for time-harmonic plane wave scattering by a single circular cylinder $\Omega_{c}$ immersed in an infinitely extending fluid medium. We consider the solution in the domain exterior to the cylinder where the field satisfies the acoustic wave equation

2.1 $$(\partial_{x}^{2}+\partial_{y}^{2}+1)\phi_{\text{ext}}=0,\;\text{for}(x,y)\in R_{2}\setminus \Omega_{c},$$

where $x=k\bar{x}$ and $y=k\bar{y}$ denote non-dimensional Cartesian coordinates, $\phi_{\text{ext}}$ is the fluid velocity potential in the exterior domain, $k=\omega \sqrt{\rho /B}$ denotes the wavenumber, ω is the angular frequency of the forcing and scattered fields, and ρ and B are the density and bulk modulus of the background medium, respectively. Here, the observed time-dependent field is given by $\text{Re}\{\phi_{\text{ext}}\exp(-\text{i}\omega t)\}$. The general solution to (2.1) takes the form

2.2 $$\phi_{\text{ext}}=\phi_{\text{inc}}+\phi_{\text{sc}}=\sum_{n=-\infty }^{\infty }\left\{a_{n}J_{n}(r)+b_{n}H_{n}^{\left(1\right)}(r)\right\}{\text{e}}^{\text{i}n\theta },$$

where $\phi_{\text{inc}}$ and $\phi_{\text{sc}}$ are the incident and scattered potentials, respectively, $a_{n}$ and $b_{n}$ refer to the as yet unknown incoming and outgoing field coefficients, respectively, $J_{n}(z)$ are Bessel functions of the first kind, $H_{n}^{\left(1\right)}(z)$ are Hankel functions of the first kind, (r,θ) is the polar form of (x,y), and we specify Neumann conditions on the walls $\partial_{r}\phi_{\text{ext}}{|}_{r=b}=0,$ with $\bar{b}$ denoting the dimensional cylinder radius (and $b=k\bar{b}$).

If we consider incident plane waves of the form $\phi_{\text{inc}}=\exp(\text{i}x\cos\theta_{\text{inc}}+\text{i}y\sin\theta_{\text{inc}})$, where $\theta_{\text{inc}}$ denotes the incident angle, then the solution is obtained straightforwardly by using a Jacobi–Anger expansion for the incident field [27, eqn. (8.511-4)]

2.3 $${\text{e}}^{\text{i}r\cos\theta }=\sum_{n=-\infty }^{\infty }{\text{i}}^{n}J_{n}(r){\text{e}}^{\text{i}n\theta },$$

and imposing the Neumann boundary condition. This gives the form

2.4 $$\phi_{\text{ext}}={\text{e}}^{\text{i}r\cos(\theta -\theta_{\text{inc}})}-\sum_{n=-\infty }^{\infty }\left\{\frac{{\text{i}}^{n}J_{n}^{\prime }(b)}{H_{n}^{(1)\prime }(b)}{\text{e}}^{-\text{i}n\theta_{\text{inc}}}\right\}H_{n}^{\left(1\right)}(r)\;{\text{e}}^{\text{i}n\theta },$$

where prime notation denotes the derivative with respect to argument, i.e. $J_{n}^{\prime }(b)=\partial_{z}J_{n}(z){|}_{z=b}$. With this canonical solution for plane wave scattering by a cylinder in mind, we now proceed to our solution outline for a cylindrical resonator.

## 3. Scattering by a single Helmholtz resonator

In order to construct a multipole solution for a single resonator at low frequencies, we combine the multipole expansion technique outlined in §2 above, with the method of matched asymptotic expansions [9,10]. This procedure is outlined in extensive detail for two-dimensional homogeneous *arrays* of resonators in earlier works by the authors [13,14]. As the inner solutions and matching procedure are unchanged from these earlier works, we outline only the updated (leading-order) outer solutions here, and simply state key results where appropriate. For reference, the acoustic wave equation (2.1) governs wave propagation in the exterior domain for all outer solutions.

### (a) Leading-order outer solution for all wall thicknesses

We begin by posing an ansatz for the exterior domain (r≥b), comprising the plane wave, a monopole source at the (small) resonator mouth, and a complete cylindrical harmonic basis satisfying the Sommerfeld radiation condition, in the form

3.1 $$\phi_{\text{ext}}={\text{e}}^{\text{i}r\cos(\theta -\theta_{\text{inc}})}+AH_{0}^{\left(1\right)}(\tilde{r})+\sum_{n=-\infty }^{\infty }c_{n}H_{n}^{\left(1\right)}(r){\text{e}}^{\text{i}n\theta },$$

where A and $c_{n}$ are as yet unknown coefficients, $\tilde{r}=\sqrt{{\left(x-x_{0}\right)}^{2}+{\left(y-y_{0}\right)}^{2}}$, and $\left(x_{0},y_{0})=(b\cos\theta_{0},b\sin\theta_{0}\right)$ lies at the midpoint of the exterior mouth. Without loss of generality, we now fix the location of the aperture by taking $\theta_{0}=0$ and consider only varying the incident angle $\theta_{\text{inc}}$ in the present work. Figure 1 presents a three-dimensional segment, and two-dimensional cross-section, of the infinitely long cylindrical Helmholtz resonator that constitutes the scatterer.

For our leading-order outer problem [9,13,14], the resonator is an *almost-closed* cylinder, and so we treat the aperture as a point source via the boundary condition [13]

3.2 $$\partial_{r}\phi_{\text{ext}}{|}_{r=b}=\frac{\text{i}A}{\pi b}\sum_{n=-\infty }^{\infty }{\text{e}}^{\text{i}n\theta },$$

which is compatible with the point source term in (3.1). After applying Graf’s addition theorem [27, eqn. (8.530)]

3.3 $$H_{0}^{\left(1\right)}(\tilde{r})=\sum_{n=-\infty }^{\infty }J_{n}(b)H_{n}^{\left(1\right)}(r){\text{e}}^{\text{i}n\theta },\;\text{for}\;r>b,$$

on the initial monopole term in (3.1), and the Jacobi–Anger expansion (2.3) on the plane wave term, we impose the Neumann condition (3.2) above to obtain

3.4 $$c_{n}=-{\text{i}}^{n}\frac{J_{n}^{\prime }(b)}{H_{n}^{(1)\prime }(b)}{\text{e}}^{-\text{i}n\theta_{\text{inc}}}-\frac{AQ_{n}}{2H_{n}^{(1)\prime }(b)},$$

where $Q_{n}=J_{n}(b)H_{n}^{(1)\prime }(b)+J_{n}^{\prime }(b)H_{n}^{\left(1\right)}(b)$. Accordingly, only the amplitude A remains unknown at this stage, and in the limit as we approach the exterior mouth we find that

3.5 $$\begin{array}{r} \lim_{r\to b}\lim_{\theta \to 0}\phi_{\text{ext}}∼{\text{e}}^{\text{i}b\cos\theta_{\text{inc}}}+\frac{2\text{i}A}{\pi }\left(\gamma_{e}-\frac{\text{i}\pi }{2}+\log\left(\frac{\tilde{r}}{2}\right)\right)-\sum_{n=-\infty }^{\infty }\left[{\text{i}}^{n}J_{n}^{\prime }(b){\text{e}}^{-\text{i}n\theta_{\text{inc}}}+\frac{AQ_{n}}{2}\right]\frac{H_{n}^{\left(1\right)}(b)}{H_{n}^{(1)\prime }(b)}, \end{array}$$

where $\gamma_{e}\approx 0.577216\ldots$ denotes the Euler–Mascheroni constant. The form of $\phi_{\text{ext}}$ in (3.5) above plays a key role in our asymptotic matching procedure, which we now briefly outline.

The method of matched asymptotic expansions refers to a powerful technique for determining approximate solutions to a wide variety of mathematical problems, including those related to wave equations [9,10], when there is a natural partitioning of the problem in terms of a suitable length scale or time scale. The method involves three crucial elements: an *outer solution*, an *inner solution*, and a matching rule. In general, for spatial problems, the outer solutions asymptotically describe the field in the far-field (i.e. away from a region with rapidly changing geometry, such as an aperture, step or inclusion), whereas inner solutions asymptotically describe near-field behaviour (e.g. in the region close to an aperture). The matching rule allows us to directly equate asymptotic expansion for the inner and outer solutions to any order. To determine the form of A in (3.1), we equate the leading order asymptotic form for the outer solution as we approach the neck from inside and outside the resonator, such as in (3.5), with the leading order inner solution as we tend to infinity in the left- and right-half planes. By equating logarithmic and non-logarithmic terms in these limits, we are able to form a system, that when solved, gives the coefficient A in closed form.

### (b) Results from asymptotic matching

For our single-resonator scattering problem, the details of the matched asymptotic procedure are very similar to that presented in earlier works [13,14] for arrays of resonators, and require only an updated outer exterior solution $\phi_{\text{ext}}$, which is given by (3.5) above. That is, the other leading-order outer solutions (i.e. for the resonator interior $\phi_{\text{int}}$ and the resonator neck $\phi_{\text{neck}}$) and both inner solutions (i.e. Φ and Ψ) are unchanged from [13,14]. After introducing the replacement for $\phi_{\text{ext}}$ (3.5) into the matching rule, we find, after considerable algebraic manipulation, that the amplitude is given by

3.6 $$A=-\frac{2\text{i}}{\pi bh_{\varepsilon }}\sum_{p=-\infty }^{\infty }\frac{{\text{i}}^{p}}{H_{p}^{(1)\prime }(b)}{\text{e}}^{-\text{i}p\theta_{\text{inc}}},$$

where

3.7 $$h_{\varepsilon }=\left\{ \begin{array}{l} \frac{4\text{i}}{\pi }\left(\gamma_{e}-\frac{\text{i}\pi }{2}+\log\left(\frac{\varepsilon }{4}\right)\right)-\frac{1}{2}\sum_{m=-\infty }^{\infty }\frac{Q_{m}^{2}}{J_{m}^{\prime }(b)H_{m}^{(1)\prime }(b)},\\ \;\text{when thin-walled},\\ \frac{4\text{i}}{\pi }\left(\gamma_{e}-\frac{\text{i}\pi }{2}+\log\left(\frac{\sqrt{q}C(q)\varepsilon }{2}\right)\right)-\frac{1}{2}\sum_{m=-\infty }^{\infty }\left\{\frac{{\overset{ˇ}{Q}}_{m}J_{m}(a)}{J_{m}^{\prime }(a)}+\frac{Q_{m}H_{m}^{\left(1\right)}(b)}{H_{m}^{(1)\prime }(b)}\right\},\\ \;\text{when moderately thick-walled},\\ \frac{2\text{i}}{\pi }\left(\gamma_{e}-\frac{\text{i}\pi }{2}-\log\left(\frac{\pi }{\varepsilon }\right)-\left[\frac{2\text{i}\tau_{3}}{\pi }+\tau_{4}\tau_{5}\right]{\left[\frac{2\text{i}\tau_{1}}{\pi }+\tau_{2}\tau_{5}\right]}^{-1}\right)-\frac{1}{2}\sum_{m=-\infty }^{\infty }\frac{Q_{m}H_{m}^{\left(1\right)}(b)}{H_{m}^{(1)\prime }(b)},\\ \;\text{when extremely thick-walled}. \end{array}\right.$$

In these expressions, we introduce the dimensionless small parameter $\varepsilon =k\bar{\ell }$, where $2\bar{\ell }$ denotes the total aperture width, $2\bar{m}$ is the total aperture length (where $m=k\bar{m}$), a=b−2m denotes the inner radius (where $a=k\bar{a}$), ${\overset{ˇ}{Q}}_{m}=J_{m}(a)H_{m}^{(1)\prime }(a)+J_{m}^{\prime }(a)H_{m}^{\left(1\right)}(a)$ and the quantity q is obtained by solving the relation [13]

3.8 $$\frac{m}{\ell }=\frac{1}{2}{\left[2E(q^{2})+(q^{2}-1)K(q^{2})\right]}^{-1}[-2E(1-q^{2})+(1+q^{2})K(1-q^{2})],$$

with $C(q)=1/\{2E(q^{2})+(q^{2}-1)K(q^{2})\},$ where E(z) and K(z) are complete elliptic integrals of the first and second kind, respectively. Furthermore, as in Smith & Abrahams [14], we define

3.9a $$\begin{array}{rl} \tau_{1} & \;=\frac{2\varepsilon }{\pi }(1-\log2)\sin(2m)-\cos(2m),\;\tau_{4}=-\frac{2\varepsilon }{\pi }(1-\log2)\sin(2m)+\cos(2m), \end{array}$$

3.9b $$\begin{array}{rl} \tau_{2} & \;=-\frac{2\varepsilon }{\pi }\sin(2m),\;\tau_{5}=\frac{2\text{i}}{\pi }\left[\gamma_{e}-\frac{\text{i}\pi }{2}-\log\left(\frac{\pi }{\varepsilon }\right)\right]-\frac{1}{2}\sum_{m=-\infty }^{\infty }\frac{{\overset{ˇ}{Q}}_{m}J_{m}(a)}{J_{m}^{\prime }(a)} \end{array}$$

3.9c $$\begin{array}{rl} \;\tau_{3} & \;=\left[\frac{2\varepsilon }{\pi }{\left(1-\log2\right)}^{2}-\frac{\pi }{2\varepsilon }\right]\sin(2m)-2(1-\log2)\cos(2m). \end{array}$$

Thus, with the form of A in (3.6) and the representation for $h_{\varepsilon }$ in (3.7), in addition to the expression for $c_{n}$ in (3.4), we have now fully prescribed $\phi_{\text{ext}}$ in (3.1) in the low-frequency asymptotic limit, for all wall thickness configurations. For reference, we also define the channel aspect ratio $h=2\bar{m}/2\bar{\ell }$ (i.e. aperture length divided by aperture width) and note that in the thin-walled limit h→0, we have q→1 and C(q)→1/2, which returns consistent expressions for $h_{\varepsilon }$ in (3.7).

Next, we examine the explicit form of the Helmholtz resonance condition for a single resonator.

### (c) Helmholtz resonance frequency

For our plane wave scattering problem, the Helmholtz resonance frequency is obtained straightforwardly by searching for the minimum value of the denominator of A in (3.6). This corresponds to the vanishing of the imaginary part of $h_{\varepsilon }$. In the limit as the wave becomes long relative to all geometric parameters, we find that

3.10 $$\lim_{\{a,b,m\}\to 0}h_{\varepsilon }\approx \left\{ \begin{array}{l} \frac{\text{i}}{\pi }\left[\frac{2}{b^{2}}-\frac{1}{4}+\gamma_{e}-\frac{\text{i}\pi }{2}+\log\left(\frac{\varepsilon^{4}}{2^{5}b^{3}}\right)\right],\\ \;\text{when thin-walled, }\\ \frac{\text{i}}{\pi }\left[\frac{2}{a^{2}}-\frac{1}{4}+\gamma_{e}-\frac{\text{i}\pi }{2}+\log\left(\frac{q^{2}C{\left(q\right)}^{4}\varepsilon^{4}}{2a^{2}b}\right)\right],\\ \;\text{when moderately thick-walled},\\ \frac{\text{i}}{\pi }\{\gamma_{e}-\frac{\text{i}\pi }{2}-\log\left(\frac{\pi^{2}b}{2\varepsilon^{2}}\right)+2\left[\frac{1}{a^{2}}-\frac{m\pi }{\varepsilon }-\frac{17}{8}-\log\left(\frac{\pi a}{2^{3}\varepsilon }\right)\right]\\ \;\times {\left[1+\frac{4\varepsilon m}{\pi }\left(\frac{1}{a^{2}}-\frac{1}{8}-\log\left(\frac{\pi a}{2\varepsilon }\right)\right)\right]}^{-1}\},\\ \;\text{when extremely thick-walled}. \end{array}\right.$$

If we then prescribe $m=\kappa_{m}\varepsilon^{\mu }$ and $a=\kappa_{a}\varepsilon^{\gamma }$ where $\kappa_{m}$ and $\kappa_{a}$ are real constants and 0<μ,γ<1, and examine the narrow aperture limit ε→0, it follows that under the dominant balance scaling μ+1−2γ>0 [14], we have

3.11 $$\begin{array}{r} h_{\varepsilon }\approx \frac{\text{i}}{\pi }\left\{\frac{2}{a^{2}}-\frac{2m\pi }{\varepsilon }-\frac{17}{4}+\gamma_{e}-\frac{\text{i}\pi }{2}+\log\left(\frac{2^{7}\varepsilon^{4}}{\pi^{4}a^{2}b}\right)\right\},\;\text{when extremely thick-walled. } \end{array}$$

Accordingly, we write the Helmholtz resonance condition in our three canonical limits as

3.12a $$\begin{array}{rl} & \frac{2}{b^{2}}-\frac{1}{4}+\gamma_{e}+\log\left(\frac{\varepsilon^{4}}{2^{5}b^{3}}\right)\approx 0,\;\text{when thin-walled, } \end{array}$$

3.12b $$\begin{array}{rl} & \frac{2}{a^{2}}-\frac{1}{4}+\gamma_{e}+\log\left(\frac{q^{2}C{\left(q\right)}^{4}\varepsilon^{4}}{2a^{2}b}\right)\approx 0,\;\text{when moderately thick-walled} \end{array}$$

3.12c $$\begin{array}{rl} \mathrm{and}\; & \frac{2}{a^{2}}-\frac{2m\pi }{\varepsilon }-\frac{17}{4}+\gamma_{e}+\log\left(\frac{2^{7}\varepsilon^{4}}{\pi^{4}a^{2}b}\right)\approx 0,\;\text{when extremely thick-walled}, \end{array}$$

and note that in all cases $h_{\varepsilon }$ takes the value 1/2 at resonance. The presence of log⁡(k) terms (implicit within the dimensionless constants) contrasts the corresponding expressions for a two-dimensional array of resonators [13,14].

The expressions in (3.12) are useful for rapidly determining the location of, or configuration for, the first Helmholtz resonance. For example, should we specify a total aperture width of $2\bar{\ell }\approx 1$ mm, which generally corresponds to a length scale at which viscous effects are negligible in acoustics, as well as a desired resonance frequency ν (e.g. ν=1 kHz, where ω=2πν or $k=18.3074\;m^{-1}$ in air), then in the thin-walled limit, we solve (3.12*a*) to find that an outer radius of $\bar{b}\approx 17.9258$ mm is required. Such values correspond to a resonator design that is readily fabricated using contemporary methods (e.g. using additive manufacturing techniques).

Note that for all configurations, the resonance condition (3.12) may conveniently be written in terms of Lambert-W functions [28], $w=W_{j}(z)$, where w satisfies wexp⁡w=z, and j=−1 or 0 for real w and z. For example, for thin-walled resonators, we have $w=-4/{\left(k\bar{b}\right)}^{2}$ and $z=-\exp\{-1/2+2\gamma_{e}+8\log(\bar{\ell }/(2\bar{b}))\}$ from (3.12*a*). On closer investigation, we find that the solution corresponding to j=0 is not relevant, as it returns an unphysically large real-valued k, for specified $\bar{\ell }$ and $\bar{b}$, hence violating the asymptotic (low-frequency) assumption underlying this work. Accordingly, the Helmholtz resonance condition may be expressed as

$$k_{\text{max}}=\frac{2}{\bar{b}}\frac{1}{\sqrt{-W_{-1}(z)}},\;\text{or}\;\omega_{\text{max}}=\frac{2}{\bar{b}}\sqrt{\frac{B}{\rho }}\frac{1}{\sqrt{-W_{-1}(z)}},\;\text{in the thin-walled case}.$$

The above result (3.12) may be extended to determine complex poles [29], which are obtained by solving for $h_{\varepsilon }=0$, see (3.10) above. For the thin-walled configuration this takes the form

$$k_{\text{max}}^{c}=\frac{2}{\bar{b}}\frac{1}{\sqrt{-W_{-1}(z^{c})}},\;\text{where}\;z^{c}=-\exp\left\{-\left(\frac{1}{2}\right)+2\gamma_{e}-\text{i}\pi +8\log\left(\frac{\bar{\ell }}{2\bar{b}}\right)\right\}.$$

Note that for complex argument $W_{j}$ has in general an infinity of roots, j∈Z; however, as already stated, the j=0 term is discarded as it violates the asymptotic assumptions, and the remaining roots (j≠0,−1) do not in fact satisfy $h_{\varepsilon }=0$ upon re-substitution. So, if we input the geometric parameters from our example above (i.e. $2\bar{\ell }\approx 1$ mm and $\bar{b}\approx 17.9258$ mm for $k=18.3074\;m^{-1}$ in air) in (3.12), we obtain $k\approx 18.2549-0.7918\text{i}\;m^{-1}$ in air, directly, corresponding to the quality factor Q=Re(k)/|2 Im(k)|≈12 meaning that, in the context of acoustics, there is strong confinement of energy for the resonator at this frequency.

## 4. Asymptotic representations of scattering coefficients

In this section, we return to the leading-order exterior field representation from §3a and examine the multipole coefficients in closer detail. We wish to obtain simple explicit expressions valid at low frequencies, i.e. for $b=k\bar{b}\ll 1$, ensuring that we preserve the partitioning ε≪b. To this end, we write the ansatz (3.1) as

4.1 $$\phi_{\text{ext}}=\phi_{\text{inc}}+\phi_{\text{sc}}={\text{e}}^{\text{i}r\cos(\theta -\theta_{\text{inc}})}+\sum_{n=-\infty }^{\infty }d_{n}H_{n}^{\left(1\right)}(r){\text{e}}^{\text{i}n\theta },$$

which arises after an application of Graf’s addition theorem (3.3), where

4.2 $$d_{n}=AJ_{n}(b)+c_{n}=\frac{\text{i}A}{\pi bH_{n}^{(1)\prime }(b)}-{\text{i}}^{n}\frac{J_{n}^{\prime }(b)}{H_{n}^{(1)\prime }(b)}{\text{e}}^{-\text{i}n\theta_{\text{inc}}}.$$

On inspection, we see that the first term in (4.2) is due to the resonator and the second term corresponds to the scattering coefficient for an ideal Neumann cylinder (2.4). Next, we truncate the sum in (4.1) so that all terms of $O(b^{4})$ are captured (i.e. we consider the orders n=−2,…,2) and presume that such a truncation is sufficient for describing the response of the resonator at low frequencies. Consequently, in the vanishing b limit, we find that

4.3a limb→0d0 ≈πib28fε[1−2fε12]−πb3cos⁡(θinc)4fε  +πib48{(1−1fε)[12−γe+iπ2−log⁡(b2)]+14−cos⁡(2θinc)2fε},

4.3b $$\begin{array}{rl} \lim_{b\to 0}d_{\pm 1} & \;\approx \mp \frac{\pi b^{2}}{4}{\text{e}}^{\mp \text{i}\theta_{\text{inc}}}\pm \frac{\pi \text{i}b^{3}}{8f_{\varepsilon }}\\ & \;\;+\frac{\pi b^{4}}{4}\left\{\pm \frac{{\text{e}}^{\mp \text{i}\theta_{\text{inc}}}}{2}\left[\frac{5}{4}+\gamma_{e}-\frac{\text{i}\pi }{2}+\log\left(\frac{b}{2}\right)\right]\mp \frac{\cos(\theta_{\text{inc}})}{f_{\varepsilon }}\right\},\;\text{and} \end{array}$$

4.3c $$\begin{array}{rl} \; & \;\lim_{b\to 0}d_{\pm 2}\approx \frac{\pi \text{i}b^{4}}{32}\left[\frac{1}{f_{\varepsilon }}-{\text{e}}^{\mp 2\text{i}\theta_{\text{inc}}}\right], \end{array}$$

where we have used the scaling $h_{\varepsilon }=4\text{i}f_{\varepsilon }/(\pi b^{2})$ and the result

4.4 $$\lim_{b\to 0}A\approx \frac{\pi \text{i}b^{2}}{4f_{\varepsilon }}(1+2\text{i}b\cos(\theta_{\text{inc}}))+\frac{\text{i}\pi b^{4}}{8f_{\varepsilon }}\left[-\frac{1}{2}+\gamma_{e}-\frac{\text{i}\pi }{2}-\cos(2\theta_{\text{inc}})+\log\left(\frac{b}{2}\right)\right].$$

With these representations in mind, we now consider crosssections for our Helmholtz resonator.

### (a) Scattering, absorption and extinction cross sections

In order to describe compactly how our resonator influences the incident plane wave, we evaluate cross-sections for the scattering, absorption and extinction strength of a given resonator. We characterize the scattering strength using the scattering cross-section for a cylinder or resonator [19, Sec. E.1]

4.5 $${\bar{\sigma }}_{\text{sc}}=\frac{4}{k}\sum_{m=-\infty }^{\infty }|d_{m}{|}^{2},$$

which we scale by the diameter of the scatterer to define the non-dimensional coefficient $Q_{\text{sc}}={\bar{\sigma }}_{\text{sc}}/(2\bar{b}),$ which is termed the *scattering efficiency* [1]. Additionally, we have the extinction cross-section

4.6 $${\bar{\sigma }}_{\text{ext}}=-\frac{4}{k}\sum_{m=-\infty }^{\infty }\text{Re}\left\{d_{m}{\text{e}}^{-\text{i}m(\pi /2-\theta_{\text{inc}})}\right\},$$

with the corresponding extinction efficiency $Q_{\text{ext}}={\bar{\sigma }}_{\text{ext}}/(2\bar{b})$. Furthermore, we note that the extinction efficiency is given by the sum [1]

4.7 $$Q_{\text{ext}}=Q_{\text{sc}}+Q_{\text{abs}},$$

where $Q_{\text{abs}}={\bar{\sigma }}_{\text{abs}}/(2\bar{b})$ denotes the absorption efficiency. Therefore, in the absence of viscosity (or other dissipative processes), we have $Q_{\text{ext}}=Q_{\text{sc}}$. Having obtained cross-section expressions in preparation for numerical investigations in §6, we now examine the impact of dissipation in thick-walled resonators.

## 5. Dissipation in extremely thick-walled resonators

In this section, we discuss the impact of incorporating viscosity within the neck region of an extremely thick-walled resonator, where a fully developed boundary layer could be expected to appear in the fluid, giving rise to viscous losses [23,24]. For thin- and moderately thick-walled resonators, boundary layer effects can be expected to be minimal, due to the smaller neck length, and so we do not consider these configurations here. To incorporate dissipative loss in a simple fashion, we specify a complex-valued wavenumber within the neck region via the replacement $k\mapsto k+\text{i}\bar{\alpha }$ where $\bar{\alpha }$ is the dimensional attenuation coefficient (units $1\;m^{-1}$). This gives rise to the outer solution in the neck (cf. [14, Eq. (2.6)])

5.1 $$\lim_{\varepsilon \to 0}\phi_{\text{neck}}∼p_{0}{\text{e}}^{\text{i}(1+\text{i}\alpha )\tilde{y}}+q_{0}{\text{e}}^{-\text{i}(1+\text{i}\alpha )\tilde{y}},$$

where $\alpha =\bar{\alpha }/k$ is the non-dimensional attenuation constant, $p_{0}$ and $q_{0}$ are unknown constants, and $\left(\tilde{x},\tilde{y})=(y,x-b\right)$ denote a local coordinate frame whose origin is centred at the exterior mouth of the resonator [14]. Using the outer solution in the exterior domain $\phi_{\text{ext}}$ (3.5), the outer solution in the neck region $\phi_{\text{neck}}$ (5.1), and the outer solution in the interior [13, eqn. (6.11)], in tandem with the inner solutions Φ and Ψ given in Smith & Abrahams [14], we obtain a result identical to (3.6) and (3.7) after asymptotic matching, but with the replacement $h_{\varepsilon }\mapsto h_{\varepsilon }^{d}$ where

5.2 $$\begin{array}{r} h_{\varepsilon }^{d}=\frac{2\text{i}}{\pi }\left(\gamma_{e}-\frac{\text{i}\pi }{2}-\log\left(\frac{\pi }{\varepsilon }\right)-\left[\frac{2\text{i}\tau_{3}^{d}}{\pi }+\tau_{4}^{d}\tau_{5}\right]{\left[\frac{2\text{i}\tau_{1}^{d}}{\pi }+\tau_{2}^{d}\tau_{5}\right]}^{-1}\right)-\frac{1}{2}\sum_{m=-\infty }^{\infty }\frac{Q_{m}H_{m}^{\left(1\right)}(b)}{H_{m}^{(1)\prime }(b)}, \end{array}$$

along with the dissipative forms

5.3a $$\begin{array}{rl} \tau_{1}^{d} & \;=\frac{2\varepsilon \gamma_{d}}{\pi }(1-\log2)\sin(2m\gamma_{d})-\cos(2m\gamma_{d}), \end{array}$$

5.3b $$\begin{array}{rl} \tau_{2}^{d} & \;=-\frac{2\varepsilon \gamma_{d}}{\pi }\sin(2m\gamma_{d}), \end{array}$$

5.3c $$\begin{array}{rl} \tau_{3}^{d} & \;=\left[\frac{2\varepsilon \gamma_{d}}{\pi }{\left(1-\log2\right)}^{2}-\frac{\pi }{2\varepsilon \gamma_{d}}\right]\sin(2m\gamma_{d})-2(1-\log2)\cos(2m\gamma_{d}),\;\text{and} \end{array}$$

5.3d $$\begin{array}{rl} \tau_{4}^{d} & \;=-\frac{2\varepsilon \gamma_{d}}{\pi }(1-\log2)\sin(2m\gamma_{d})+\cos(2m\gamma_{d}), \end{array}$$

in which $\gamma_{d}=1+\text{i}\alpha$. Presuming that α is small (i.e. $\alpha ∼O(\varepsilon^{0})$), we have that

5.4 $$\begin{array}{rl} \lim_{\{a,b,m\}\to 0}h_{\varepsilon }^{d} & \;\approx \frac{2\text{i}}{\pi }\{\frac{\gamma_{e}}{2}-\frac{\text{i}\pi }{4}-\frac{1}{2}\log\left(\frac{\pi^{2}b}{2\varepsilon^{2}}\right)+[\frac{1}{a^{2}}-\frac{m\pi }{\varepsilon }-\frac{2\pi m^{3}\gamma_{d}^{2}}{3\varepsilon }\\ & \;-\frac{2m^{2}\gamma_{d}^{2}}{a^{2}}-\frac{17}{8}-\log\left(\frac{\pi a}{8\varepsilon }\right)]\cdot {\left[1-2m^{2}\gamma_{d}^{2}+\frac{4\varepsilon m\gamma_{d}^{2}}{\pi a^{2}}\right]}^{-1}\}, \end{array}$$

which, under the μ+1−2γ>0 dominant balance limit from §3c, takes the form

5.5 $$h_{\varepsilon }^{d}\approx \frac{\text{i}}{\pi }\left\{\gamma_{e}-\frac{\text{i}\pi }{2}-\frac{17}{4}+\frac{2}{a^{2}}-\frac{2m\pi }{\varepsilon }+\log\left(\frac{2^{7}\varepsilon^{4}}{\pi^{4}a^{2}b}\right)-4m^{2}\gamma_{d}^{2}\left[\frac{1}{a^{2}}+\frac{m\pi }{3\varepsilon }\right]\right\}.$$

Thus, the Helmholtz resonance condition is given by

5.6 $$\frac{2}{a^{2}}-\frac{2m\pi }{\varepsilon }-\frac{17}{4}+\gamma_{e}+\log\left(\frac{2^{7}\varepsilon^{4}}{\pi^{4}a^{2}b}\right)-4m^{2}(1-\alpha^{2})\left[\frac{1}{a^{2}}+\frac{m\pi }{3\varepsilon }\right]=0.$$

Accordingly, by substituting (5.6) into (5.5), we find that *at* the Helmholtz resonance, we have

5.7 $$h_{\varepsilon }^{d}\approx \frac{1}{2}+\frac{8}{\pi }\alpha m^{2}\left[\frac{1}{a^{2}}+\frac{m\pi }{3\varepsilon }\right].$$

From the definition of A in (3.6) (with the replacement $h_{\varepsilon }\mapsto h_{\varepsilon }^{d}$), we see that the presence of dissipation acts to lower the amplitude of the monopole source when on-resonance. That is, $h_{\varepsilon }^{d}$ increases from the value of 1/2 observed in the lossless case to (5.7). For reference, we use $h_{\varepsilon }^{d}=4\text{i}f_{\varepsilon }^{d}/(\pi b^{2})$ for the replacement $f_{\varepsilon }\mapsto f_{\varepsilon }^{d}$. Furthermore, the above forms for $h_{\varepsilon }^{d}$ (5.2) and $\tau_{j}^{d}$ (5.3) correctly tend to the earlier nondissipative results for $h_{\varepsilon }$ (3.7) and $\tau_{j}$ (3.9) in the limit of vanishing loss α→0.

## 6. Numerical results

In this section, we compute a selection of scattered field profiles $\phi_{\text{sc}}$ for representative resonators from all three wall thickness configurations, numerically evaluate a selection of scattering, absorption and extinction efficiencies as a function of non-dimensionalized frequency (wavenumber), and examine how well the asymptotic representations for $d_{n}$ in (4.3) perform relative to the full multipole forms (4.2). Additionally, we consider the impact of dissipative loss (from a boundary layer in the neck) on all cross-sections for a representative resonator in the extremely thick-walled limit. In all examples, we consider air as the background medium, possessing a bulk modulus B=141.83 KPa and density $\rho =1.2041\;\mathrm{kg}\;m^{3}$, although this is done without loss of generality.

**Figure 2.**
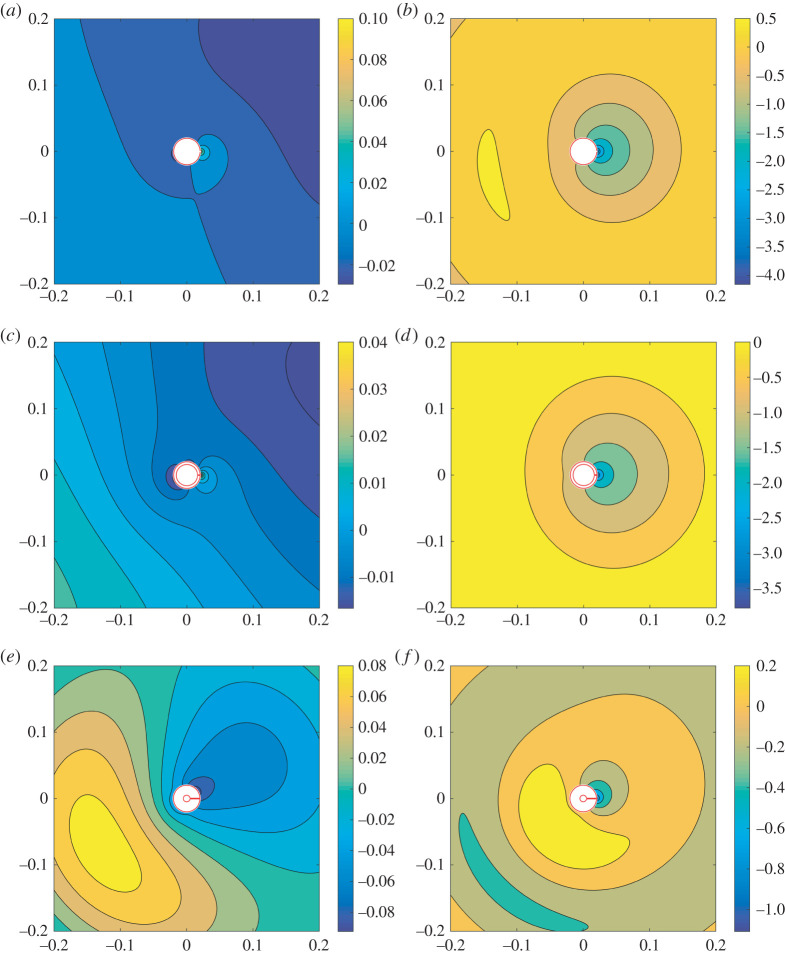
Scattered field profiles $\phi_{\text{sc}}$ (3.1) for (*a*,*b*) a thin-walled resonator at $k=k_{H}/2$ and $k=k_{H}\approx 16.2136\;m^{-1}$, respectively, where $k_{H}$ satisfies the Helmholtz resonance condition (3.12*a*); (*c*,*d*) a moderately thick-walled resonator ($h=2\bar{m}/2\bar{\ell }=4$) at $k_{H}/2$ and $k_{H}\approx 13.3001\;m^{-1}$ respectively, where $k_{H}$ satisfies (3.12*b*); (*e*,*f*) an extremely thick-walled resonator (h=15) at $k_{H}/2$ and $k_{H}\approx 26.9376\;m^{-1}$, respectively, where $k_{H}$ satisfies (3.12*c*). In all figures, we use $\bar{b}=20\;\mathrm{mm}$, $2\bar{\ell }=1\;\mathrm{mm}$ and $\theta_{\text{inc}}=\pi /6$. (Online version in colour.)

In figure 2, we present the scattered field $\phi_{\text{sc}}$ for a representative thin-walled, moderately thick-walled, and extremely thick-walled resonator, where all resonators possess the same outer radius $\bar{b}$ and aperture width $2\bar{\ell }$. In each setting, we examine the scattered response at the first Helmholtz resonance frequency, $k_{H}$, satisfying the relevant condition in (3.12), and at the low frequency $k_{H}/2$ (i.e. away from the resonance frequency), to consider how the field profile is modified as we approach the resonance. In the thin-walled case, we observe a field enhancement of over two orders of magnitude in the transition from $k=k_{H}/2$ to $k=k_{H}$, with $\phi_{\text{sc}}$ taking a maximum value at the resonator mouth. A similar behaviour is observed for the moderately thick-walled case (h=4), although we find that the field enhancement is approximately halved. This reduction continues for the extremely thick-walled resonator where the field enhancement is now slightly over one order of magnitude, and is likely due to the fact that the volume inside the resonator decreases, since we increase the aperture neck length $2\bar{m}$ while keeping the outer radius $\bar{b}$ constant. Accordingly, to achieve the strongest field enhancements, we advise that the resonator wall thickness be taken as thin as possible. Additionally, the strongest backscattering is observed in the thin- and extremely thick-walled representative configurations. For reference, a plane wave of incidence angle $\theta_{\text{inc}}=\pi /6$ is considered in all cases.

**Figure 3.**
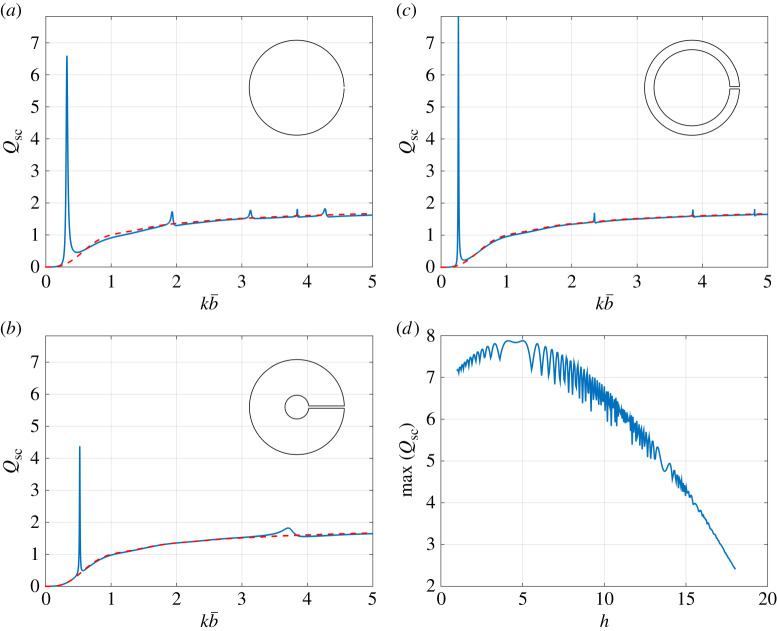
Scattering efficiency $Q_{\text{sc}}$ (from (4.5)) versus (scaled) frequency for a (*a*) thin-walled resonator, (*b*) moderately thick-walled resonator (h=4), and (*c*) extremely thick-walled resonator (h=15); (*d*) gives the maximum value for $Q_{\text{sc}}$ at the first Helmholtz resonance versus the channel aspect ratio $h=2\bar{m}/2\bar{\ell }$ (with results obtained using the thick-walled formulation). In all figures, we use $\bar{b}=20\;\mathrm{mm}$, $2\bar{\ell }=1\;\mathrm{mm}$ and $\theta_{\mathrm{inc}}=\pi /6$, and results for a single closed Neumann cylinder of the same radius are superposed for reference (dashed red). Inset figures: resonator geometry (not to scale). (Online version in colour.)

In figure 3, we compute the scattering efficiency $Q_{\text{sc}}$ (following the definition for the scattering cross-section ${\bar{\sigma }}_{\text{sc}}$ in (4.5)) for the three resonator configurations considered in figure 2. For all $Q_{\text{sc}}$ figures, we superpose the result for a closed Neumann cylinder for reference (dashed red curves). We find that for all resonator configurations, a considerable enhancement is observed in the scattering efficiency at the first Helmholtz resonance frequency $k_{H}$ (obtained by solving (3.12) for each geometry), and also observe that the $Q_{\text{sc}}$ curves tend to that of a closed Neumann cylinder away from the higher-frequency resonance peaks. These higher frequency peaks are associated with the fundamental modes of a closed Neumann cylinder, i.e. $J_{n}^{\prime }(a)=0$ for all n, and with the fundamental modes of the neck region, in the case of thick-walled resonators. As the wall thickness increases, we observe that the spacing between Helmholtz resonances increases (as the enclosed internal resonator area becomes smaller); however, we also find that the peak scattering efficiency $\max(Q_{\text{sc}})$ does not exhibit an obvious trend. To investigate this behaviour, we plot $\max(Q_{\text{sc}})$ against the channel aspect ratio h in figure 3*d*, where we find that a maximum scattering efficiency, for our chosen outer radius and aperture width, occurs at h≈4.11 and h≈5.01. The oscillations in the $\max(Q_{\text{sc}})$ curve are due to phase cancellation effects within the neck (see $\phi_{\text{neck}}$ in (5.1) with α=0), where the outward and inward propagating wave components of the solution either destructively or constructively interfere with each other.

**Figure 4.**
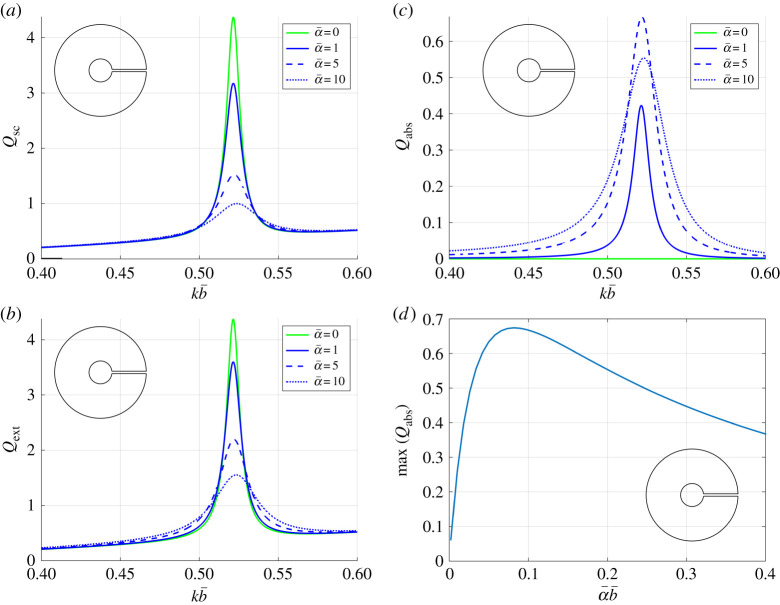
The impact of introducing dissipation in the neck region of an extremely thick-walled resonator, via the attenuation coefficient $\bar{\alpha }$: (*a*) scattering efficiency $Q_{\text{sc}}$ (defined from (4.5)), (*b*) absorption efficiency $Q_{\text{abs}}$ (defined from (4.7)), and (*c*) extinction efficiency $Q_{\text{ext}}$ (defined from (4.6)), versus the (scaled) frequency; (*d*) gives the maximum value for $Q_{\text{abs}}$ at the first Helmholtz resonance versus the (scaled) attenuation coefficient $\bar{\alpha }$. In all figures, we use $\bar{b}=20$ mm, $2\bar{\ell }=1$ mm, h=15 and $\theta_{\text{inc}}=\pi /6$. Inset figures: resonator geometry (not to scale). (Online version in colour.)

In figure 4, we examine the impact of introducing a boundary layer in the neck of our representative extremely thick-walled resonator (h=15) from figure 2 near the first Helmholtz resonance. We compute the scattering, absorption and extinction efficiencies for a range of attenuation values $\bar{\alpha }$, observing that in the limit as $\bar{\alpha }\to \infty$, results for the scattering and extinction efficiency coefficients tend to the results for (lossless) Neumann cylinders straightforwardly (see the dashed red curve in figure 3*c* for reference). Such behaviour is expected due to the increasing resistance in the neck. However, the absorption efficiency coefficient is found first to rise with increasing loss, and then to decrease monotonically towards zero. The presence of this maximum $Q_{\text{abs}}$ therefore suggests a range of validity for $\bar{\alpha }$ in our treatment (and resonator geometry), i.e. $0<\bar{\alpha }\bar{b}<0.08$ when considering dissipative loss. In general, we advise that only α values that lie below this peak should be considered as physically reliable. For all attenuation values, we observe that scattering is the dominant process in the extinction of the incident power, with the reduction in $Q_{\text{ext}}$ being driven by the reduction in $Q_{\text{sc}}$.

**Figure 5.**
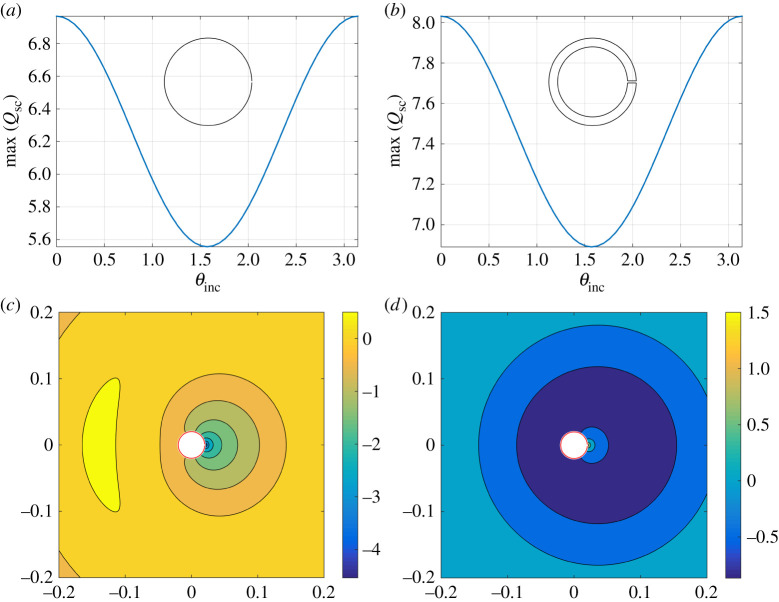
The impact of incident angle $\theta_{\text{inc}}$ on the maximum (lossless) scattering efficiency $\max(Q_{\text{sc}})$ (defined from (4.5)), for a (*a*) thin-walled and (*b*) moderately thick-walled (h=4) resonator. Inset figures: resonator geometry (not to scale). Corresponding scattered field profiles $\phi_{\text{sc}}$ (3.1) for the thin-walled resonator configuration in figure 5*a* at $k=k_{H}=16.2136$ m${\;}^{-1}$ for (*c*) $\theta_{\text{inc}}=0$ and (*d*) $\theta_{\text{inc}}=\pi$. Here, we use $\bar{b}=20$ mm and $2\bar{\ell }=1$ mm where applicable. (Online version in colour.)

In figure 5, we examine how the scattering efficiency $Q_{\text{sc}}$ at the first Helmholtz resonance is impacted by the incident angle of the incoming plane wave. We present results for a thin- and moderately thick-walled resonator (h=4) showing that an absolute maximum scattering efficiency is observed at incidence angles along the mirror plane for the resonator (recall that the aperture is located at $\theta_{0}=0$), with a minimum observed in the orthogonal direction at $\theta_{\text{inc}}=\pi /2$. That is, the incident wave does not have to be directed into the resonator $\theta_{\text{inc}}=\pi$ in order to achieve maximal scattering efficiency, as the same result is obtained for $\theta_{\text{inc}}=0$. An identical behaviour is observed for extremely thick resonator configurations and so we do not present the corresponding figure here. Figure 5*c*,*d* presents the scattered field $\phi_{\text{sc}}$ for the thin-walled resonator at the Helmholtz resonance frequency $k=k_{H}=16.2136\;m^{-1}$ for the incidence angles $\theta_{\text{inc}}=0$ and $\theta_{\text{inc}}=\pi$, respectively, demonstrating how very different field profiles can still return identical scattering cross-sections (i.e. an identical amount of incident power scattered).

**Figure 6.**
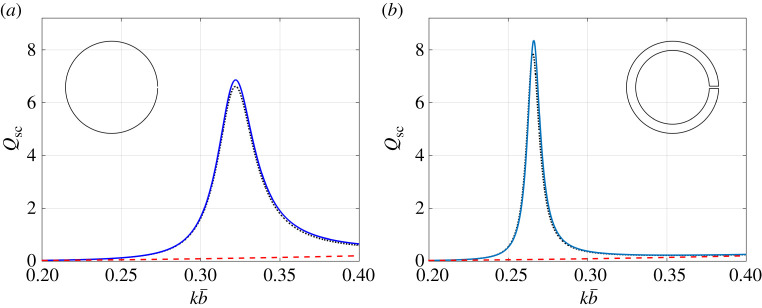
Comparing the scattering efficiency $Q_{\text{sc}}$ (defined from (4.5)), evaluated using the full (4.2) (dotted black line) and asymptotic forms (4.3) (blue line) for $d_{n}$ corresponding to (*a*) a thin-walled resonator, with the asymptotic form for $h_{\varepsilon }$ (3.10) taken to $O(b^{4})$, and (*b*) moderately thick-walled (h=4) resonator. In these figures, we use $\bar{b}=20$ mm, $2\bar{\ell }=1$ mm and $\theta_{\text{inc}}=\pi$ where applicable; the result for a single closed Neumann cylinder of the same radius is superposed for reference (dashed red). Inset figure: resonator geometry (not to scale). (Online version in colour.)

In figure 6, we consider the full multipolar (4.2) and asymptotic (4.3) forms for $d_{n}$ when evaluating the (lossless) scattering efficiency $Q_{\text{sc}}$. Results are given for the thin-walled and moderately thick-walled (h=4) resonator geometries considered in figure 2. In general, we find that the asymptotic estimates (4.3) work well for frequencies below the first Helmholtz resonance $k_{H}$. However, for the thin-walled geometry, asymptotic forms for $h_{\varepsilon }$ (3.10) must be taken to $O(b^{4})$ in order to recover the $Q_{\text{sc}}$ peak as shown (this representation of $h_{\varepsilon }$ is not presented here for compactness). Additionally, results for the extremely thick-walled (h=15) configuration are not presented here as we require asymptotic forms for $h_{\varepsilon }$ and $d_{0,\pm 1,\pm 2}$ to a very high order in b for accuracy, although for $k\ll k_{H}$, we find that the asymptotic forms (4.3) for $d_{n}$ work well for all resonator configurations.

For reference, numerical investigations examining the impact of varying the channel aspect ratio h and the aperture half-angle $\theta_{\text{ap}}$ on the first Helmholtz resonance frequency $k_{H}$ for an extremely thick-walled resonator (h=15) return a similar qualitative behaviour to that seen in Smith & Abrahams [14] for two-dimensional arrays. Additionally, the cross-section results for lossless cylinders match those presented in Rubinow & Wu [30] after a factor 2 correction in their definition is taken into account.

## 7. Discussion

In this paper, we have presented an analytic solution method for deducing plane wave scattering by a single two-dimensional Helmholtz resonator. Our solution procedure, valid at low frequencies, combines multipole methods with the method of matched asymptotic expansions, extending results from earlier works by the authors on homogeneous two-dimensional arrays of resonators [13,14]. In addition to describing the scattered field, we determine the extinction, absorption and scattering cross-sections for a selection of resonator designs and compare these against results for an isolated Neumann cylinder.

Numerical investigations demonstrate considerable field enhancement near the resonator mouth, at the first Helmholtz resonance frequency, with a strong dependence on the wall thickness. We have found that optimal wall thicknesses exist to achieve maximal cross-sections (efficiencies) for a prescribed outer radius and incidence angle. Furthermore, we consider the impact of a boundary layer emerging in the resonator neck, giving rise to viscous dissipative losses. Although a lossy neck gives rise to moderate values for the absorption efficiency, the corresponding reduction in the scattering efficiency has the net effect of diminishing the extinction efficiency at the first Helmholtz resonance and beyond, where in the limit of large loss, we find that all cross-sections return to the results for a Neumann cylinder, as expected. In general, the maximal scattering efficiency $Q_{\text{sc}}$ is found for incidence wave angles that lie along the mirror symmetry plane for the resonator, despite the fact that very different scattered field profiles are observed for $\theta_{\text{inc}}=0$ and $\theta_{\text{inc}}=\pi$. The formulation presented here should prove useful for ongoing theoretical and experimental work by other groups [15,31,32].

Another formalism for describing wave scattering by objects is the resonance scattering theory (RST) framework [33–35]. This involves expanding the scattered field in terms of a sum of singularities in the complex frequency domain. Our scattered field representation is the standard one, involving sums of Bessel functions, except for the one pole closest to the real line corresponding to the contribution from the resonator (as discussed in the text surrounding (3.12)). This captures the Helmholtz resonance accurately and is akin to a hybrid RST-multipole treatment.

Recently, Melnikov *et al.* [15] have conducted experimental and theoretical work on plane wave scattering by a single Helmholtz resonator of the type considered here. In their work, they use a *lumped-element model* [23] to describe the resonator response which takes the form of a third-order ordinary differential equation (a nonlinear spring model). Such phenomenological-type modelling is not required to determine the plane wave scattering response of a single resonator, as we have shown here. That is, the asymptotic matching procedure recovers field representations that are theoretically indistinguishable from the genuine field at low frequencies. Furthermore, Melnikov *et al.* [15] construct an acoustic analogue to the electric *polarizability tensor* in electrostatics [3], with non-zero off-diagonal terms that are referred to as elements of a Willis coupling tensor (see appendix A in the present work for an in-depth discussion). However, we stress that Willis coupling in general refers to the tensors that emerge within generalized constitutive relations for effective structured media, and furthermore, as shown in earlier work on two-dimensional arrays of resonators by the authors [14], that anisotropy and not bianisotropy is observed in bulk. Accordingly, the presence of Willis-like behaviour (depolarizability effects) in the acoustic polarizability tensor [15], as shown in our appendix, does not necessarily correlate with an effective Willis tensor effect in bulk. Although Willis coupling tensors are vanishing at low frequencies (for centrosymmetric unit cells), they are still nonetheless present at low frequencies, and given their absence in the effective dispersion equation in bulk [13,14], we therefore do not find evidence that two-dimensional arrays of single-aperture Helmholtz resonators exhibit bianisotropy (Willis coupling).

In addition to how this work may relate to Willis coupling, future work includes careful parameter sweeps to examine the impact of aperture width on all cross-sections, as well as studies considering scattering by random suspensions of resonators, finite clusters and one-dimensional arrays (with the latter currently under investigation by the authors). The impact of different internal resonator geometries (e.g. square) may be of interest. Although the results derived here are formally derived for small aperture widths $2\bar{\ell }$, our treatment appears to hold for much wider apertures than those considered numerically in the present work (see [13,14]). Other future work includes an extension of our model to incorporate nonlinear effects in the fluid within the neck, which removes the need to assume the existence of nonlinearity *a priori*, as is required for lumped element model treatments.

## Figures and Tables

**Figure 7.**
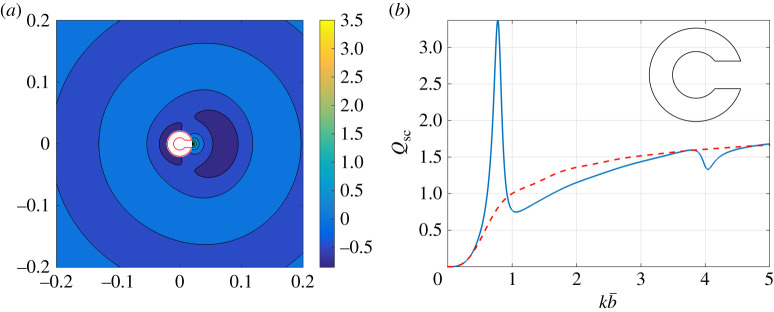
Reference calculations for the configuration considered in Melnikov *et al.* [15]: (*a*) the scattered potential $\phi_{\text{sc}}$ as defined in (3.1) at $k=k_{H}=40.3589\;{\text{m}}^{-1}$ and (*b*) the (lossless) scattering efficiency $Q_{\text{sc}}$ (defined from (4.5)). In both figures, we use $\bar{b}=20$ mm, $2\bar{\ell }=12$ mm, $h=2\bar{m}/2\bar{\ell }=5/6$ and $\theta_{\text{inc}}=\pi$; the result for a single closed Neumann cylinder of the same radius is superposed for reference (dashed red). Inset figure: resonator geometry (not to scale). (Online version in colour.)

## Data Availability

This article has no additional data.

## Appendix A. Distinguishing acoustic depolarizability from Willis coupling

When designing a periodically structured acoustic material, it is important to consider the possibility that the resulting metamaterial exhibits generalized constitutive behaviour at low frequencies, even if entirely composed of materials satisfying conventional constitutive laws. In the literature, such a generalized response is known as bi-anisotropy or Willis coupling [36]. It is also possible that the metamaterial may exhibit enhanced nonlinear constitutive behaviour [37], but we do not consider that possibility here. Recently, a selection of works [15,31,32] have claimed that an analogue to Willis coupling is exhibited in the *scattering response* of a single meta-atom, which we now examine.

In order to understand the Willis-like coupling effect exhibited by a single resonator, we follow the procedure in Quan [32] and express the (dimensional) scattered pressure for a single resonator, embedded in a uniform background fluid, in terms of an ideal (point-source) monopole and dipole at the origin, i.e. we write the scattered pressure in the form

A 1 $${\bar{P}}_{\text{sc}}=-\text{i}k^{2}\frac{c_{p}^{2}}{4}MH_{0}^{\left(1\right)}(k\bar{r})-\text{i}\frac{k^{3}c_{p}^{2}}{4}(D_{\bar{x}}\cos\theta +D_{\bar{y}}\sin\theta )H_{1}^{\left(1\right)}(k\bar{r}),$$

where $\bar{r}$ denotes the dimensional distance from the origin, $c_{p}=\sqrt{B/\rho }$ is the free-space phase velocity, M is the acoustic monopole amplitude and $\left(D_{\bar{x}},D_{\bar{y}}\right)$ are the acoustic dipole amplitudes. Note that overbar notation generally denotes dimensional quantities, although this is not used for constitutive parameters. Through the use of orthogonality relations it follows that [32, Eq.(2.51)]

A 2 $$\begin{array}{rl} & M=\frac{2\text{i}c_{p}^{-2}}{\pi k^{2}H_{0}^{\left(1\right)}(k\bar{r})}\int_{0}^{2\pi }{\bar{P}}_{\text{sc}}(\bar{r},\theta )\;\text{d}\theta \\ \mathrm{and}\;\; & \left\{\begin{array}{c} D_{\bar{x}}\\ D_{\bar{y}} \end{array}\right\}=\frac{4\text{i}c_{p}^{-2}}{\pi k^{3}H_{1}^{\left(1\right)}(k\bar{r})}\int_{0}^{2\pi }{\bar{P}}_{\text{sc}}(\bar{r},\theta )\left\{\begin{array}{c} \cos(\theta )\\ \sin(\theta ) \end{array}\right\}\;\text{d}\theta , \end{array}\}$$

where ${\bar{P}}_{\text{sc}}=\text{i}\omega \rho {\bar{\phi }}_{\text{sc}}$ in turn follows from the linearized form of Bernoulli’s law [23]. Thus, using the dimensionless form of $\phi_{\text{sc}}$ given in (4.1), we are able to write (A 2) in the form

A 3 $$\left[\begin{array}{c} M\\ D_{\bar{x}}\\ D_{\bar{y}} \end{array}\right]=\left[\begin{array}{ccc} \alpha_{11} & \alpha_{12} & 0\\ \alpha_{21} & \alpha_{22} & 0\\ 0 & 0 & \alpha_{33} \end{array}\right]\left[\begin{array}{c} {\bar{P}}_{\text{inc}}^{\left(0\right)}\\ {\bar{v}}_{\text{inc}}^{\left(0\right)}\\ {\bar{w}}_{\text{inc}}^{\left(0\right)} \end{array}\right],$$

where

A 4a $$\begin{array}{rl} & \begin{array}{r} \alpha_{11}=\frac{4\text{i}}{{\left(c_{p}k\right)}^{2}}\left(\frac{2h_{\varepsilon }^{-1}}{{\left[\pi k\bar{b}H_{0}^{(1)\prime }(k\bar{b})\right]}^{2}}-\frac{J_{0}^{\prime }(k\bar{b})}{H_{0}^{(1)\prime }(k\bar{b})}\right),\;\alpha_{12}=-\frac{16\rho h_{\varepsilon }^{-1}}{\pi^{2}c_{p}k^{4}{\bar{b}}^{2}H_{0}^{(1)\prime }(k\bar{b})H_{1}^{(1)\prime }(k\bar{b})}, \end{array} \end{array}$$

A 4b $$\begin{array}{rl} & \begin{array}{r} \alpha_{21}=\frac{16\text{i}h_{\varepsilon }^{-1}}{\pi^{2}k^{5}{\bar{b}}^{2}c_{p}^{2}H_{0}^{(1)\prime }(k\bar{b})H_{1}^{(1)\prime }(k\bar{b})},\;\alpha_{22}=\frac{8\rho }{k^{3}c_{p}}\frac{J_{1}^{\prime }(k\bar{b})}{H_{1}^{(1)\prime }(k\bar{b})}-\frac{32\rho h_{\varepsilon }^{-1}}{c_{p}k^{3}{\left[\pi k\bar{b}H_{1}^{(1)\prime }(k\bar{b})\right]}^{2}} \end{array}, \end{array}$$

A 4c $$\begin{array}{rl} \mathrm{and}\;\; & \alpha_{33}=\frac{8\rho }{k^{3}c_{p}}\frac{J_{1}^{\prime }(k\bar{b})}{H_{1}^{(1)\prime }(k\bar{b})}, \end{array}$$

with ${\bar{P}}_{\text{inc}}^{\left(0\right)}={\bar{P}}_{\text{inc}}(\bar{r}=0)=\text{i}kc_{p}\rho$ denoting the incident pressure, and the vector $\left({\bar{v}}_{\text{inc}}^{\left(0\right)},{\bar{w}}_{\text{inc}}^{\left(0\right)})=(\partial_{\bar{x}}{\bar{\phi }}_{\text{inc}}{|}_{\bar{r}=0},\partial_{\bar{y}}{\bar{\phi }}_{\text{inc}}{|}_{\bar{r}=0})=\text{i}k(\cos(\theta_{\text{inc}}),\sin(\theta_{\text{inc}})\right)$ representing the incident velocity, at the origin. In the limit as b→0, we find that

A 5a $$\alpha_{11}∼\frac{2\text{i}}{{\left(kc_{p}\right)}^{2}h_{\varepsilon }}\left[-1+{\left(k\bar{b}\right)}^{2}\left(\frac{1}{2}-\gamma_{e}+\frac{\text{i}\pi }{2}-\log\left(\frac{k\bar{b}}{2}\right)\right)\right]-\frac{\pi \text{i}{\left(k\bar{b}\right)}^{2}}{4},\;\alpha_{12}∼\frac{4\rho \bar{b}}{c_{p}kh_{\varepsilon }}$$

and

A 5b $$\alpha_{21}∼-\frac{4\text{i}\bar{b}}{c_{p}^{2}k^{2}h_{\varepsilon }},\;\alpha_{22}∼-\frac{2\pi \text{i}\rho {\bar{b}}^{2}}{c_{p}k}+\frac{8\rho {\bar{b}}^{2}}{c_{p}kh_{\varepsilon }},\;\alpha_{33}∼-\frac{2\pi \text{i}\rho {\bar{b}}^{2}}{c_{p}k}.$$

Thus, if we were to describe our meta-atom in terms of an ideal point-source monopole and dipole placed at the origin, we would find that the presence of the aperture (i.e. to create a resonator) causes these amplitudes to depend upon both the incident pressure and the incident velocity defined at the origin. Such cross-coupling is termed Willis coupling by some authors, and is absent in the expressions for ideal Neumann cylinders: this is readily seen in the aperture closing limit ε→0 where $h_{\varepsilon }\to -\text{i}\infty$ in (A 4), which then returns $\alpha_{12}=\alpha_{21}=0$ and $\alpha_{22}=\alpha_{33}$ [15, S59,S64]. In order to fully appreciate the relationship between the response of a single meta-atom (A 3) and the response of a bulk acoustic metamaterial made from an array of meta-atoms, we now examine the bulk properties of a genuine Willis fluid for reference purposes.

Within the acoustics and fluids community, Willis coupling effects are not so widely known, and so we briefly outline the derivation here, starting with the conservation of mass and momentum equations [32,36]

A 6 $$\partial_{t}\varepsilon -\bar{\nabla }\cdot \bar{\text{u}}=0\;\text{and}\;\partial_{t}{\bar{\text{p}}}_{a}+\bar{\nabla }\bar{P}=0,$$

where $\varepsilon =-(\text{trace}(\rho )/\text{trace}(\rho_{0})-1)$ is the scalar volumetric strain, ρ and $\rho_{0}$ denote the dynamic mass density tensor (of rank two) and the density at rest, respectively, $\bar{\text{u}}$ is the particle velocity, ${\bar{\text{p}}}_{a}$ is the acoustic momentum density, $\bar{\nabla }$ is the gradient vector and $\bar{P}$ is the pressure. For a uniform Willis fluid, we have the generalized constitutive relations (frequency domain form)

A 7 $${\bar{\text{p}}}_{a}=\rho \bar{\text{u}}-\eta \bar{P}\;\text{and}\;\varepsilon =-B^{-1}\bar{P}+\gamma \cdot \bar{\text{u}},$$

where η and γ are Willis tensors (of rank one). Substituting these constitutive relations (A 7) into the conservation equations (A 6), and eliminating $\bar{\text{u}}$ through differentiation and substitution, we obtain the acoustic wave equation for a Willis fluid in the form

A 8 $$B^{-1}\partial_{t}^{2}\bar{P}+\bar{\nabla }\cdot [\rho^{-1}(\eta \partial_{t}\bar{P}-\bar{\nabla }\bar{P})]-\gamma \cdot \partial_{t}[\rho^{-1}(\eta \partial_{t}\bar{P}-\bar{\nabla }\bar{P})]=0.$$

As a simplifying example, if we consider plane wave solutions of the form $\exp(\text{i}{\bar{k}}_{B}\bar{x}-\text{i}\omega t)$ in *an acoustic metamaterial* said to exhibit Willis coupling effects (i.e. $\{\eta =\eta_{\mathrm{eff}}$, $\gamma =\gamma_{\mathrm{eff}}\}\neq 0$), and which conveniently possesses an isotropic density $\rho =\rho_{\mathrm{eff}}\text{I}$, then we expect to find a dispersion equation arising from (A 8) of the form

A 9 $${\bar{k}}_{B}^{2}+\omega {\bar{k}}_{B}[{\left(\eta_{x}\right)}_{\text{eff}}+{\left(\gamma_{x}\right)}_{\text{eff}}]-\omega^{2}(\rho_{\text{eff}}B_{\text{eff}}^{-1}-\eta_{\text{eff}}\cdot \gamma_{\text{eff}})=0,$$

where the subscript eff denotes effective quantities and ${\bar{\text{k}}}_{B}$ denotes the dimensional Bloch vector. The key point is that the dispersion equation for a Willis material features $\omega^{2}$, $\omega {\bar{k}}_{B}$ and ${\bar{k}}_{B}^{2}$ terms. For reference, the expression (A 9) is identical to the one-dimensional form given in Quan [32, eqn. (2.68)] with the replacements $\gamma_{\text{eff}}\mapsto -\gamma_{\text{eff}}$ and $\eta_{\text{eff}}\mapsto -\eta_{\text{eff}}$.

Having determined both a representative dispersion equation for an acoustic metamaterial exhibiting Willis coupling in bulk (A 9), and the amplitude-pressure-velocity relationship for a single meta-atom (A 3), we now turn to results from earlier works by the authors and others [14,38]: these show that the dispersion equation for a *two-dimensional square array* of meta-atoms (cylindrical Helmholtz resonators) possesses the structure ${\bar{k}}_{Bi}{\left(\rho_{ij}^{\text{eff}}\right)}^{-1}{\bar{k}}_{Bj}-\omega^{2}B_{\text{eff}}^{-1}=0$ at low frequencies, which is identical to that for linear uniform anisotropic media. In light of these observations, we do not find evidence to suggest that *two-dimensional arrays* of Helmholtz resonators possess low-frequency Willis coupling effects in bulk, despite the presence of Willis-like effects in the scattering response of a single meta-atom. How this effect is averaged out in this limit certainly warrants further investigation; however, it seemingly does not manifest in bulk at low frequencies and so we encourage the use of alternative nomenclature (e.g. Willis-like depolarizability) for the behaviour observed in (A 3).

As a final corollary, an apparent Willis coupling effect could inadvertently be introduced where it does not genuinely exist, through a specific decomposition of $B_{\text{eff}}^{-1}$ or $\rho_{\text{eff}}$ to recover the form of (A 7). This is due to the fact that uniqueness is not present in any homogenization description, in the absence of additional constraints. For the purposes of comparison, we present the scattered field profile $\phi_{\text{sc}}$ and scattering efficiency $Q_{\text{sc}}$ for the resonator geometry considered in Melnikov *et al.* [15] in figure 7. Here, we observe relatively strong enhancements in both the scattered field and scattering efficiency at the Helmholtz resonance $k_{H}$, as expected, and only comment that there could be room for obtaining a stronger response through an optimization search of the type considered in figure 3*d*.
